# Kidney, ureter, and urinary bladder segmentation based on non-contrast enhanced computed tomography images using modified U-Net

**DOI:** 10.1038/s41598-024-66045-6

**Published:** 2024-07-03

**Authors:** Dong-Hyun Jang, Juncheol Lee, Young-Jin Jeon, Young Eun Yoon, Hyungwoo Ahn, Bo-Kyeong Kang, Won Seok Choi, Jaehoon Oh, Dong Keon Lee

**Affiliations:** 1 Department of Public Healthcare Service, Seoul National University Bundang Hospital, Seongnam, Republic of Korea; 2 Department of Emergency Medicine, College of Medicine, Hanyang University, 222 Wangsimni-ro, Seongdong-gu, Seoul, 04763 Republic of Korea; 3 AIDOT Inc., Seoul, Republic of Korea; 4 Department of Urology, College of Medicine, Hanyang University, Seoul, Republic of Korea; 5 Department of Radiology, Seoul National University Bundang Hospital, Seongnam, Republic of Korea; 6 Department of Radiology, College of Medicine, Hanyang University, Seoul, Republic of Korea; 7 Department of Emergency Medicine, Seoul National University Bundang Hospital, 13620, 82, Gumi-ro 173 Beon-gil, Bundang-gu, Seongnam-si, Gyeonggi-do Republic of Korea; 8 Department of Emergency Medicine, Seoul National University College of Medicine, Seoul, Republic of Korea

**Keywords:** Urogenital diseases, Urinary tract

## Abstract

This study was performed to segment the urinary system as the basis for diagnosing urinary system diseases on non-contrast computed tomography (CT). This study was conducted with images obtained between January 2016 and December 2020. During the study period, non-contrast abdominopelvic CT scans of patients and diagnosed and treated with urinary stones at the emergency departments of two institutions were collected. Region of interest extraction was first performed, and urinary system segmentation was performed using a modified U-Net. Thereafter, fivefold cross-validation was performed to evaluate the robustness of the model performance. In fivefold cross-validation results of the segmentation of the urinary system, the average dice coefficient was 0.8673, and the dice coefficients for each class (kidney, ureter, and urinary bladder) were 0.9651, 0.7172, and 0.9196, respectively. In the test dataset, the average dice coefficient of best performing model in fivefold cross validation for whole urinary system was 0.8623, and the dice coefficients for each class (kidney, ureter, and urinary bladder) were 0.9613, 0.7225, and 0.9032, respectively. The segmentation of the urinary system using the modified U-Net proposed in this study could be the basis for the detection of kidney, ureter, and urinary bladder lesions, such as stones and tumours, through machine learning.

## Introduction

Urinary stone is a condition in which deposits that are not properly excreted from the kidneys crystallise in the urinary tract and cause obstruction. It is one of the most common causes of urinary tract obstruction^1,2^. Abdominopelvic computed tomography (CT) is the modality of choice to diagnose and establish a treatment plan by simultaneously providing information on the location and size of the stones^3^.

Urinary stones are often relatively small, and various calcified structures may resemble urinary stones in the abdominal or pelvic cavity. Accordingly, for accurate diagnosis of urinary stones on abdominopelvic CT, segmentation of the urinary system (consisting of the kidney, ureter, and urinary bladder), from other adjacent structures should be performed, rather than simply finding a structure that resembles a stone. However, accurately segmenting a ureter that is small and often runs close to other intra-abdominal structures, such as vascular structures that look similar, is sometimes time-consuming and challenging.

With the development of image processing technology through machine learning, various models have been developed to automatically segment organs and make specific diagnoses using deep learning techniques with medical images. Recent advances in computer processing have made it possible to evaluate an entire CT scan consisting of multiple images beyond a single X-ray or a specific image extracted from CT using a deep learning technique^4,5^. Regarding urinary stones, several studies have processed images using deep learning techniques, although most of them have been limited to the kidney or have used contrast images^6–8^.

Few studies have evaluated the accuracy of segmentation of the entire urinary system, through deep learning techniques in a non-contrast CT image, which cannot distinguish adjacent structures through varying degrees of enhancement. The purpose of this study was to create a model for segmentation of the urinary system consisting of the kidney; proximal, middle, and distal ureters; and urinary bladder on non-contrast abdominopelvic CT as the basis for diagnosing urinary system diseases using a neural network model and to evaluate its performance.

## Results

**Figure 1:**
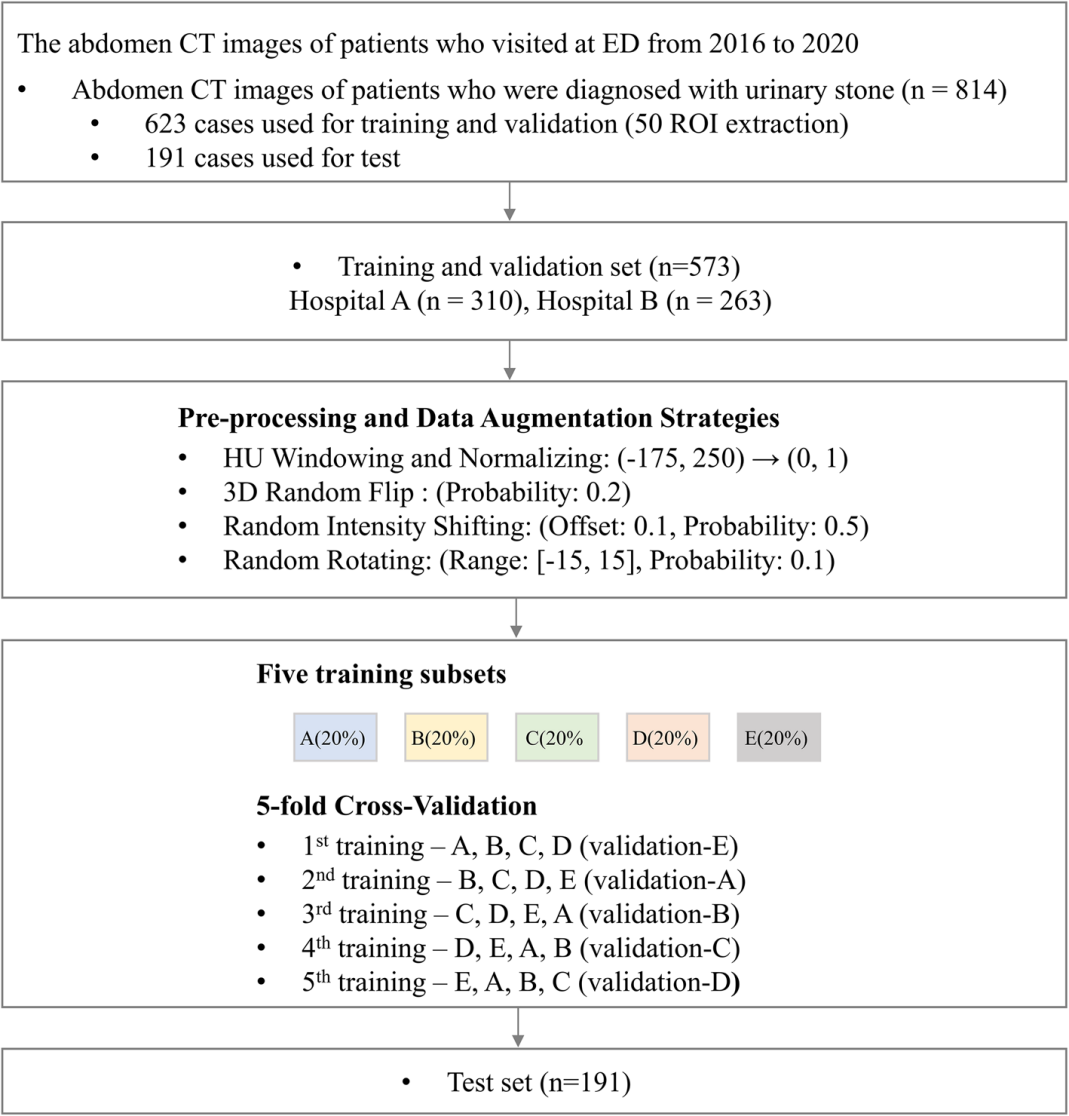
Flow chart of data collection, image processing, and analysis for urinary system segmentation. *CT* computed tomography, *ED* emergency department, *ROI* region of interest.

A total of 814 CT images were collected: 623 male abdominopelvic images for training and validation, and 191 images from both male and female patients for testing. A fivefold cross-validation (8:2) was applied to 573 cases, except for 50 used for training the region of interest (ROI) extraction (Fig. 1). The clinical characteristics of the training and validation datasets, as well as the test dataset, are presented in Table 1.

**Table 1:** Baseline characteristics of enrolled patients.

	Training and validation dataset (n = 573)	Test dataset (n = 191)
Age of patients	50.4 ± 14.4	48.83 ± 14.29
Male sex (%)	573 (100)	156 (81.7)
Number of stones		
1	291 (50.8)	105(34.9)
2	118 (20.6)	60(19.9)
3	52 (9.1)	22(7.3)
4	40 (7.0)	4(1.3)
≥ 5	72 (12.5)	0
Size of the largest stone		
< 4 mm	57 (9.9)	95(49.7)
≥ 4 mm, < 10 mm	222 (38.8)	73(38.2)
≥ 10 mm	294 (51.3)	3(1.6)
Location of the largest stone		
Right side	275 (48.0)	148(49.2)
Kidney	93 (16.2)	144(47.8)
Proximal ureter	252 (44.0)	82(27.2)
Middle ureter	66 (11.5)	15(5.0)
Distal ureter	129 (22.5)	57(18.9)
Urinary bladder	33 (5.8)	3(1.0)

Categorical and continuous variables are represented by a number (%) and mean ± standard deviation, respectively.

**Figure 2:**
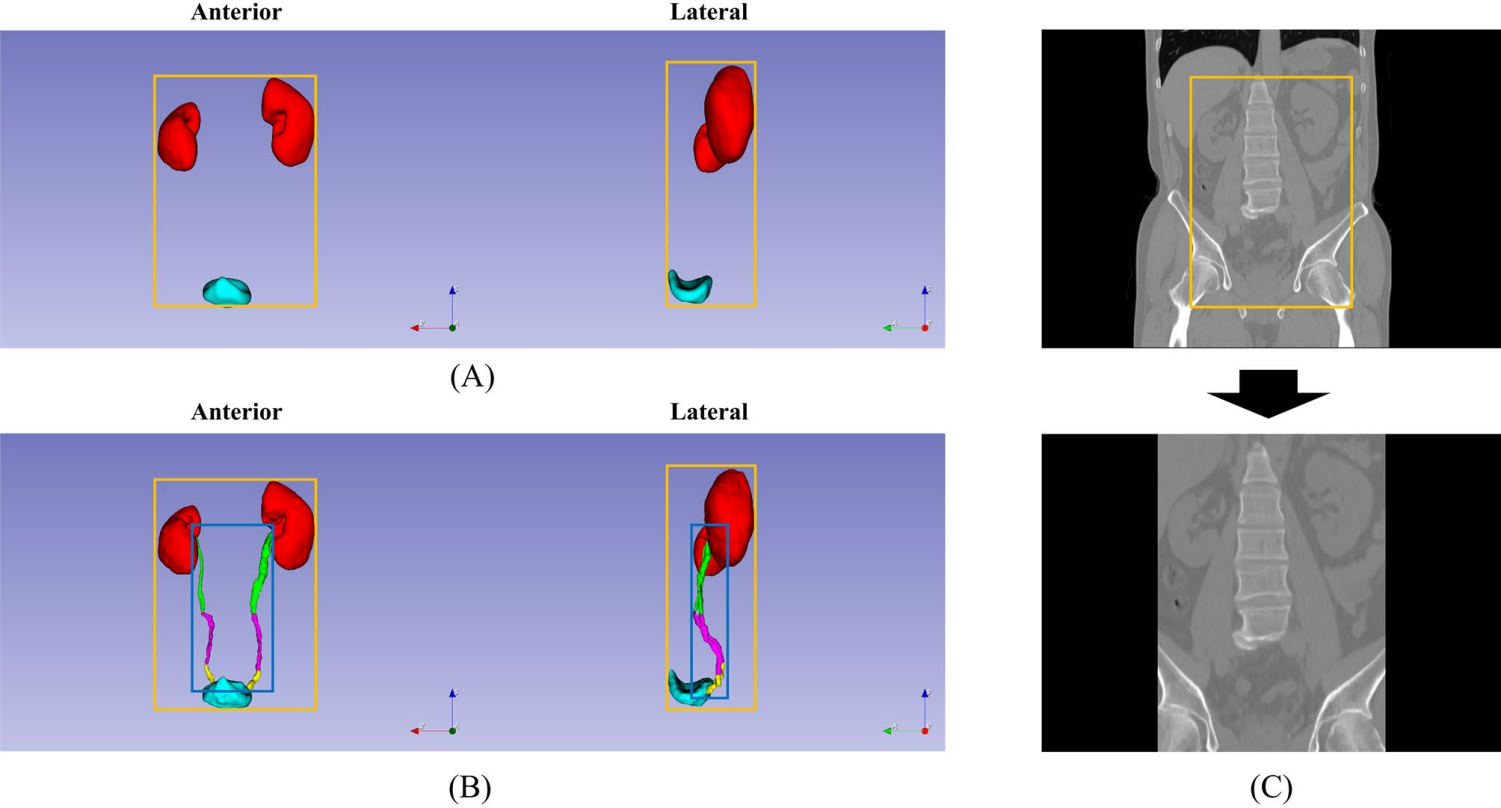
Representative results of region of interest extraction. (**A**) Prediction results from anterior and lateral perspectives using a modified U-Net model in step 1. (**B**) Predicted region of interest and ground truth of the urinary system from anterior and lateral perspectives. The orange box refers to the ROI extracted through the two kidneys and urinary bladder, and the blue box refers to the area where the ureter is present in the image. (**C**) ROI extraction is additionally cropped by 15 mm (superior and inferior directions) and 6 mm (all the other directions). *ROI* region of interest.

The results of ROI extraction are summarised in Table 2 and Fig. 2. During ROI extraction, the average Dice score for the kidney and urinary bladder was 0.9483. For the 623 CT images used in this study, none of the ureters travelled outside the cuboid determined by the two kidneys and bladder in the 3D space.

**Table 2:** Dice score of region of interest (ROI) extraction and cross-validation results in urinary system segmentation.

	Avg	Kidney	Ureter				Urinary bladder
			Total ureter	Proximal ureter	Middle ureter	Distal ureter	
ROI extraction	0.9483 (± 0.0211)	0.9632 (± 0.0412)					0.9334 (± 0.0432)
Segmentation							
Avg (95% CI)	0.8673 (± 0.0055)	0.9651 (± 0.0019)	0.7172 (± 0.0124)	0.7165 (± 0.0158)	0.6677 (± 0.0143)	0.6489 (± 0.0203)	0.9196 (± 0.0072)
Split-1	0.8687 (± 0.1360)	0.9624 (± 0.0448)	0.7127 (± 0.1408)	0.7228 (± 0.1183)	0.6557 (± 0.0987)	0.6323 (± 0.1046)	0.9311 (± 0.0405)
Split-2	0.8749 (± 0.1253)	0.9669 (± 0.0528)	0.7322 (± 0.1482)	0.7255 (± 0.1265)	0.6826 (± 0.1025)	0.6702 (± 0.1119)	0.9255 (± 0.0485)
Split-3	0.8576 (± 0.1427)	0.9641 (± 0.0498)	0.6954 (± 0.1460)	0.6854 (± 0.1192)	0.6466 (± 0.1034)	0.6213 (± 0.1082)	0.9133 (± 0.0466)
Split-4	0.8687 (± 0.1273)	0.9677 (± 0.0531)	0.7251 (± 0.1415)	0.7179 (± 0.1184)	0.6835 (± 0.1074)	0.6742 (± 0.1080)	0.9133 (± 0.04911)
Split-5	0.8666 (± 0.1289)	0.9645 (± 0.0460)	0.7206 (± 0.1376)	0.7308 (± 0.1279)	0.6703 (± 0.1249)	0.6465 (± 0.1187)	0.9146 (± 0.0426)

**Figure 3:**
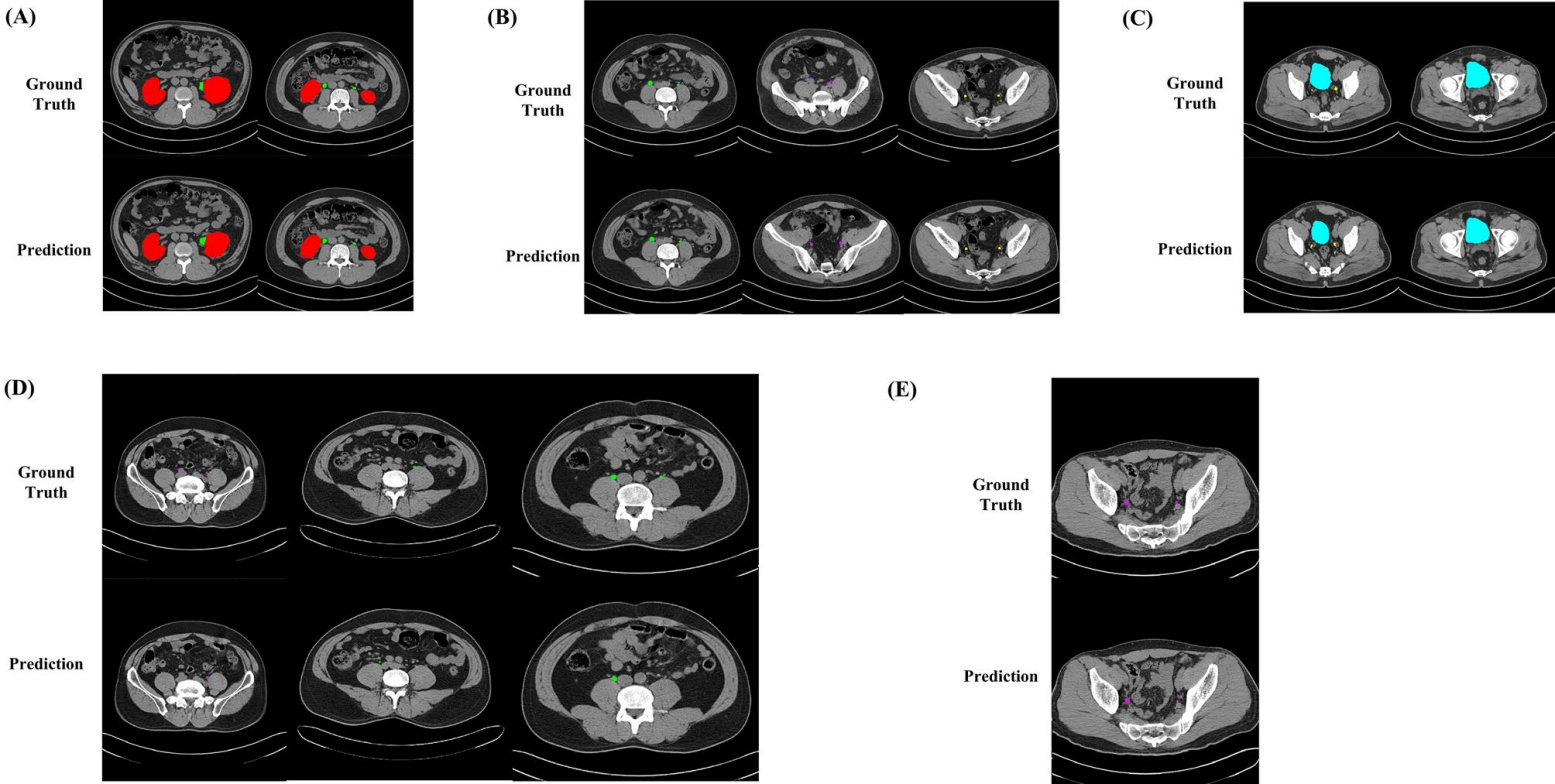
Representative examples of accurate and inaccurate urinary system segmentation. Each second row presents the prediction result by the model, and the first row presents the corresponding ground truth. (**A**) Accurate cases for the kidney and corresponding ground truth. (**B**) Accurate cases for the proximal, middle, and distal ureters and each corresponding ground truth. (**C**) Accurate cases for the urinary bladder and corresponding ground truth. (**D**) Inaccurate cases for the ureters and each corresponding ground truth. Failure to perform segmentation due to the proximity of the ureter to surrounding muscle or vascular structures, as observed in the ground truth. (**E**) Inaccurate case for the ureters and each corresponding ground truth. Erroneously segments adjacent vascular structure as the ureter.

Figure 3 shows the outcomes of restoring the urinary system segmentation results to the space of the original image for correctly segmented and inaccurately segmented cases, respectively. The segmentation results for the kidney and urinary bladder were generally good, with only minor inaccuracies in the margins due to the difficulty in distinguishing the boundaries between these organs and other solid organs on non-contrast CT images. However, in the ureter segmentation results, more concerning errors could be seen, such as incorrectly segmenting or failing to segment the ureter. These errors primarily occurred when the ureter ran closely intertwined with the surrounding muscle or vascular structures. Table 2 shows the fivefold cross-validation and the test results of the segmentation of the urinary system. In the fivefold cross validation, the average Dice coefficient for the whole urinary system was 0.8673. The average Dice coefficient of the best performing model was 0.8749, and the Dice coefficients for each class (kidney, ureter, and urinary bladder) were 0.9669, 0.7322, and 0.9255, respectively. In the test dataset, the average Dice coefficient of best performing model for whole urinary system was 0.8623, and the Dice coefficients for each class (kidney, ureter, and urinary bladder) were 0.9613, 0.7225, and 0.9032, respectively (Table 3).

**Table 3:** Comparison of Dice scores for urinary system segmentation between the proposed modified U-Net model, U-net with various components, and 2D based models in state of the art with simple multi-class implementation.

	Avg	Kidney	Ureter				Urinary bladder
			Total ureter	Proximal ureter	Middle ureter	Distal ureter	
Proposed modified U-net	0.8623 (± 0.0289)	0.9613 (± 0.0044)	0.7225 (± 0.1584)	0.7121 (± 0.1286)	0.6558 (± 0.1228)	0.6085 (± 0.1235)	0.9032 (± 0.0579)
U-Net model							
Baseline	0.7835	0.8751	0.57	0.5875	0.5427	0.5515	0.9055
With transposed convolution	0.8021	0.907	0.6012	0.6124	0.595	0.6004	0.8982
With multi-segmentation decoder	0.815	0.9315	0.5998	0.6134	0.5885	0.598	0.9137
With ROI extraction and slice windowing	0.831	0.9376	0.6309	0.6385	0.6163	0.6005	0.9243
Other 2D based models in state of the art							
UNet++	0.7964	0.914	0.5774	0.5888	0.5698	0.5589	0.898
PraNet	0.8208	0.9392	0.6501	0.6762	0.6487	0.6087	0.8732
nnU-Net	0.8332	0.9423	0.6573	0.6838	0.631	0.6163	0.8999
Swin-UNet	0.8481	0.9488	0.6924	0.6974	0.6462	0.673	0.9031

Table 3 shows the Dice scores for the segmentation of the urinary system in each region for several previously proposed models and the modified U-Net model proposed in this study. From the dataset used in this study, the modified U-Net model achieved higher Dice scores than the other models for all the regions of the urinary system.

## Discussion

In our study, we proposed a modified U-Net model, in which the kidney, ureter (proximal, middle, and distal), and urinary bladder were segmented in two steps using ROI extraction and urinary system segmentation. We analysed the Dice coefficient for each part in test dataset, and the Dice coefficients of our model for the kidney, proximal ureter, middle ureter, distal ureter, and urinary bladder were 0.9613, 0.7121, 0.6558, 0.6085, and 0.9032, respectively.

In medical images, various organs can be distinguished based on their anatomical positions and characteristic textures. For congenital anomalies or diseases causing structural abnormalities, the estimated origin is determined based on the previously known pathophysiology, and the diagnosis is made accordingly. Therefore, to accurately diagnose a disease that occurs in a specific organ, an accurate segmentation of the organ must be performed when there is an anatomical change caused by the disease, as well as in the case of normal anatomy.

Recently, to help diagnose diseases in the urinary system, several studies have been performed to segment the kidney and urinary bladder by radiography, CT, or magnetic resonance imaging using machine learning techniques. da Cruz et al. proposed an experiment using Alexnet and U-Net-based architectures for segmentation of the kidneys from CT scans and reported a Dice score of 0.9517 from validation with 90 CT images after training on 210 CT images^9^. Ma et al. applied the U-Net model to urinary bladder segmentation from a dataset consisting of 173 CT scans. They reported an average Jaccard index of 82.6%^10^.

Additionally, several studies have reported the performance of tracking and segmenting ureter travel using CT and radiography. Hadjiiski et al. attempted to track the ureter with CT urography images with contrast media using a combined model-guided pathfinding analysis and segmentation system, and completely succeeded in tracking 120 (97%) of 124 ureters^11^. Rani et al. proposed the KUB-UNet model and identified the optimum loss function and batch size to improve its performance^12^. They reported maximum values of the Dice coefficient of 0.7966, 0.6474, and 0.8992 when performed on the testing dataset for the kidney, ureters, and urinary bladder, respectively. These previous studies showed that it is possible to segment the urinary system on contrast images with high accuracy.

However, in a study using images obtained after administration of contrast media, such as urography, the urinary system or nearby organs can be distinguished by a difference in the degree of enhancement by contrast media^13,14^. Especially in urography analysis, where the urinary system is well differentiated by the contrast agent, it is possible that the difference in enhancement between the urinary system and adjacent organs had a greater effect on the segmentation performance than the characteristics of the anatomical structure of the urinary system.

In patients with diseases of the urinary system, contrast imaging, such as CT urography, is not always possible. In some patients with impaired renal function, contrast administration may be restricted; therefore, non-contrast CT is preferred^15^. Moreover, regarding urinary stone diagnosis and treatment planning, most guidelines recommend non-contrast CT as a modality of choice^3,16^. Although segmenting the urinary system and locating urinary stones on non-contrast CT images have been attempted, these tasks have been confined to the kidney rather than the entire urinary system^6–8^. However, a crucial point here is that most urinary stones requiring urgent treatment are in the ureter^3,16^.

In this study, the Dice coefficients of the kidney and urinary bladder were high, whereas the Dice coefficient of the ureter was low. In particular, the Dice scores of the middle and distal ureters were lower than those of the proximal ureters. The ureter was as small as 3–4 mm on the CT axial scan, and the middle and distal ureters are located close to the blood vessels and adnexa. In lean patients, particularly with a small amount of intra-abdominal fat, it can be difficult to separate those structures and identify the distal ureter^17^. Therefore, there may be numerous instances in which it is difficult for radiologists to trace the ureter from the proximal to the distal end. It may not be disputed that the Dice score of the ureter is lower than that of a large kidney or urinary bladder.

Furthermore, the average Dice coefficient for the ureter analysed as a whole ureter, which includes the proximal, middle, and distal ureter subclasses, was higher than the scores for these subclasses analysed separately. This is because the ureteric subclasses overlapped at the boundaries, affecting each other's Dice score.

We conducted a deep learning CT study of patients with urolithiasis. A stone in the urinary system can cause hydronephrosis due to an obstructive effect, resulting in a larger than normal ureter diameter. A ureter with a large diameter can be advantageous for ureter segmentation training because it is simple to trace the entire ureter. Furthermore, because most ureter stones manifest only on one side, our deep learning model trained segmentation not only for ureters with a larger diameter but also for ureters of normal size. Urinary stone disease patients often have stones in only one kidney or ureter. Therefore, the CT images we utilised included both urinary systems without disease and with urinary stone disease. As a result of our study, the segmentation results were promising in this dataset. And, since the focus of this study was to develop a model to segment the urinary system, automatic localization or size measurement of urinary tract stones was not conducted. Considering several previous studies that have measured encrustation on stents inserted into the ureter, it is believed that further research using this segmentation model to accurately diagnose the location and size of stones in urinary stone disease may be feasible^18,19^.

The challenging experiment in this study had the following strengths. Firstly, a novel approach to multi-segmentation decoding is introduced in our study, where a binary segmentation model is extended to accommodate multi-class segmentation tasks. This involves the incorporation of individual decoders, each capable of handling distinct features generated by the encoder. Secondly, a transposed convolution is employed during the upsampling phase of the decoder in the proposed model, effectively reducing potential information loss associated with interpolation. Thirdly, a preprocessing technique is introduced in both the training and inference phases of our model. Randomised cropping is employed for ROI extraction as an augmentation strategy during the training phase, thereby contributing to enhancing the model's robustness and accuracy during inference. Furthermore, the adoption of a sliding window during inference is implemented to reduce memory overhead, while also allowing the model to concentrate on finer details within small objects, such as the ureter, even at higher resolutions. Applying a multi-segmentation decoder, transposed convolution, and ROI extraction and slice windowing to U-net seems to improve performance by enhancing the model's ability to accurately predict small anatomical structures such as ureters. The proposed model with all three of these features outperformed the other models, including UNet++, PraNet, nnU-Net, Swin-UNet with our dataset (Table 3).

The main purpose of the study was to evaluate the performance of the modified U-Net itself. Therefore, in this study, various post-processing methods were not explored; instead, the process that is considered standard was the only one employed. However, it is possible that better segmentation performance could be achieved by refining the predictions through the application of appropriate post-processing techniques. Possible post-processing techniques include morphological operations such as erosion and dilation and conditional random fields, and the effectiveness of these techniques requires further investigation.

This study was conducted using only CT obtained from male patients for the training and validation process. In human anatomy, there is little difference between men and women in the upper pelvis with respect to the organs within the abdomen, including the urinary system^20^. Therefore, it seems unlikely that using only male CT would have caused bias regarding the kidney and ureter located above the pelvis. However, the structure within the pelvic cavity shows some differences in anatomy due to the difference of reproductive organs^21^. Female reproductive organs in the pelvis have a complex structure compared to males, and they can be difficult to distinguish from the distal ureter or urinary bladder in CT images occasionally. Therefore, we used only CT scans of male patients to reduce the controversy over ground truth labelling for segmentation. Despite training and validating our model on CT scans of male patients only, it demonstrated promising performance on a test dataset that included CT scans of female patients. However, excluding CT scans of female patients during the training process could be one of the major limitations in our study.

This study has some limitations. First, we did not perform abdominal CT in women for training and validation. Second, we conducted a multicentre study, although the data on abdominopelvic CT for patients with ureter stones from two tertiary centres and our proposed model might not be suitable for other hospitals. Third, images of urinary systems with anomalies or with only a single kidney were excluded from the analysis. Finally, in our study, only two-dimensional CT was analysed. Since 3D-based models require more computational resources and parameters compared to 2D-based models^22,23^, the 2D-based model to detect small objects such as the ureter at high resolution was chosen instead of opting for the 3D-based model. Further study is needed with employing 3D-based models to investigate the possibility of enhancing prediction of the continuity and correlation between slices could be achieved.

In conclusion, our proposed modified U-Net deep learning model demonstrated decent performance for kidney, ureter, and urinary bladder segmentation. The segmentation modified U-Net performed herein could be the basis for the detection of kidney, ureter, and urinary bladder lesions, such as stones and tumours, by machine learning.

## Methods

### Data collection

This multi-centre retrospective study was conducted using non-contrast abdominopelvic CT images obtained at two tertiary academic hospitals (Seoul and Gyeonggi-do, Republic of Korea) between August 2021 and November 2021. During the study period, non-contrast abdominopelvic CT scans of adult male patients treated and diagnosed with urolithiasis in the emergency departments of the two institutions between January 2016 and December 2020 were collected for training and validation dataset. The CT scans collected from one institution had a slice gap of 5 mm and a slice thickness of 5 mm, and those collected from another institution had a slice gap of 3 mm and a slice thickness of 4 mm. The CT scans of patients with congenital anomalies of the urinary system, with a catheter inserted (double J catheter or percutaneous nephrostomy), or with only one kidney, were excluded from the study. After the completion of the training and validation process, additional CT scans were collected to evaluate the performance of the proposed model. Additional CT scans corresponding to approximately 30% of the training and validation dataset were collected regardless of the sex of the patients, applying the same inclusion and exclusion criteria over the same period.

The CT images were collected using the Picture Archiving and Communication System (PACS, INFINITT Healthcare, Seoul, Republic of Korea) in the Digital Imaging and Communication in Medicine (DICOM) format. During data collection, all personally identifiable information included in the CT images was deleted. Instead, an arbitrary coded number was assigned to each CT scan and managed accordingly. Among the CT scans collected, 50 cases were randomly selected and used to train the extraction of the ROI, and the remaining images were used to train and validate the performance of urinary system segmentation.

Labelling for ground truth segmentation masking of the urinary system was performed using AVIEW Modeller software (Corelinesoft, Seoul, Republic of Korea). Before the ground truth segmentation process, all researchers involved in the ground truth creation underwent a review course conducted by a uroradiologist. The course focused on the identification of the urinary system and accurate tracking of the ureter on non-contrast CT images. The initial ground truth segmentation of the urinary system on CT images was performed by six radiologic technologists. Each technologist was assigned a subset of CT images with a similar number for segmentation. During the process, the technologists were instructed to mark images for reconfirmation if they were uncertain about the ground truth. Images with completed initial ground truth segmentation were then distributed to four emergency physicians and three radiologists, all of whom had over seven years of clinical experience and were actively practising. They reviewed the images for reconfirmation and made corrections as needed. In cases where even these experts found it challenging to accurately locate the urinary system, a uroradiologist was consulted for further guidance and correction. The kidney, part of the proximal ureter, and urinary bladder were automatically identified by radiodensity differences between the internal organs and adjacent fat tissue, and incorrectly selected margins were manually corrected. The ureter was then manually segmented while checking the travel of the ureter from the renal pelvis to the urinary bladder. The ureter was divided into proximal, middle, and distal ureters, based on the upper and lower margins of the sacroiliac joint. The proximal ureter was defined as from the ureteropelvic junction to the level of the superior margin of the sacroiliac joint, the middle ureter was defined as between the superior and inferior margins of the sacroiliac joint, and the distal ureter was defined as from the level of the inferior margin of the sacroiliac joint to the urinary bladder wall. During manual segmentation of the urinary system, the number, location, and size of urinary stones were also identified within the CT images to characterise urinary stone disease in the included CT images. The analysis of the stone was done in the same manner as the segmentation process. Initial evaluation was done by a radiologic technologist, followed by confirmation by emergency physicians and radiologists. Segmentation and labelling of each part of the urinary system was stored in the Neuroimaging Informatics Technology Initiative (NIfTI) format.

This study was approved by the Institutional Review Boards of Seoul National University Bundang Hospital (B-2108-705-107) and Hanyang University Hospital (HYUH 2021-04-090-001), and the requirement for informed consent was waived. This study was conducted in accordance with the Checklist for Artificial Intelligence in Medical Imaging (CLAIM)^24^, and all study procedures were conducted in accordance with the Declaration of Helsinki.

### Data preprocessing and augmentation

The CT images express brightness with a precision of a 16-bit integer and are between −1024 and 3072. However, in a typical image deep learning model, it is common to receive floating-type pixel values between 0 and 1 as input. Therefore, when CT images are used for deep learning, their intensity must be adjusted. Normalisation is performed on intensity values within the range of [−175, 250] to [0, 1]. The value used as the basis for scaling was the target value set to better represent the region of interest.

Next, simple augmentation methods were used to create an abundant dataset. First, random flips applied to each axis were attempted with a probability of 0.2. Second, a random intensity shift that gave each offset and probability of 0.1 and 0.5 was applied. Then, a random rotation with a probability of 0.1 and a range of magnitudes with -15, 15 was used.

### Modified U-Net

The algorithm designed for urinary system segmentation in the present study was based on U-Net, widely used for image segmentation in biomedical image processing^25^. Among the elements that comprise the urinary system, segmentation of the ureters on non-contrast abdominopelvic CT images is particularly difficult because of their small size (1–10 pixels) and similar radiodensity as adjacent blood vessels or solid organs.

**Figure 4:**
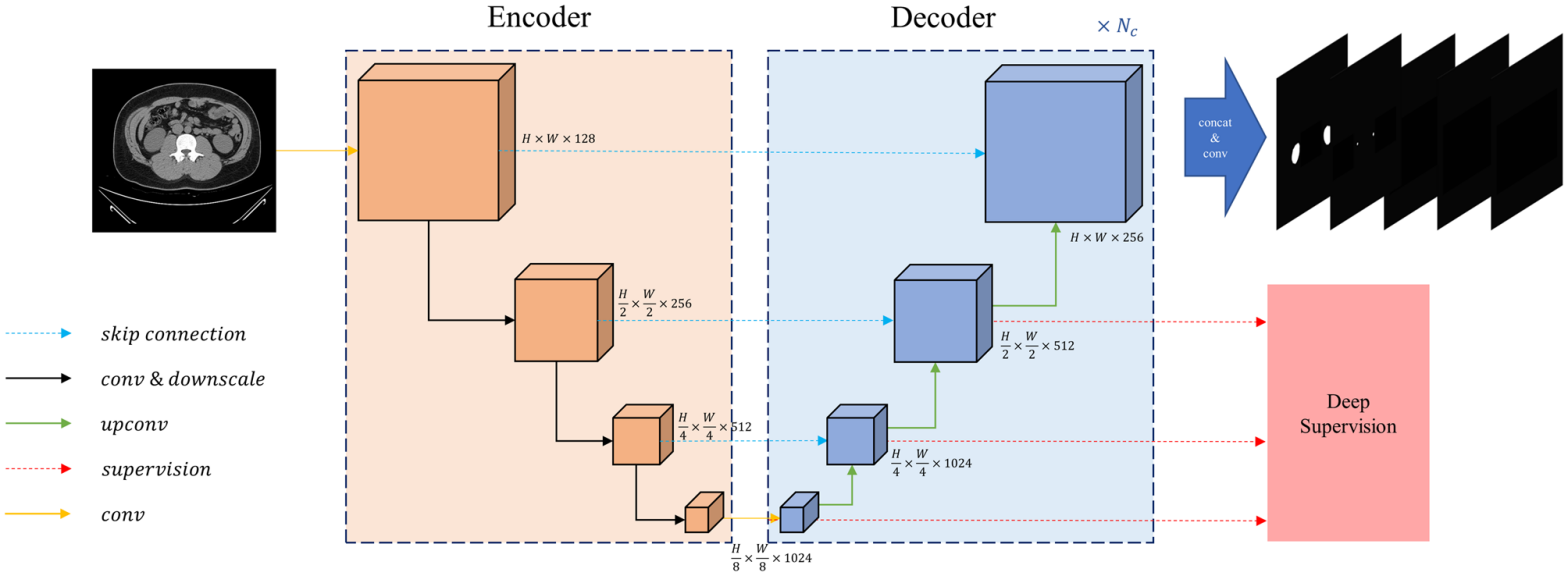
Modified U-Net architecture. The architecture not only maintains the resolution of the input but also enables multi-class prediction by configuring as many decoders in parallel as N_c_. The output is configured with the same size as the input size.

The architecture of the modified U-Net proposed in this study is shown in Fig. 4. The encoder consists of four layers. To extract feature maps from different scales, the size of the feature map gradually decreases as the layers progress. In general, methods commonly used in medical segmentation can be easily adapted to support multi-class segmentation by simply adding channels to the final layer^26,27^. In contrast, the proposed algorithm attempted to interpret the anatomical structure of organs with different characteristics by constructing number of decoders (N_c_) of the same structure. The decoder is configured to have a one-to-one correspondence with the encoder, and a skip connection is introduced between each pair of encoder and decoder layers to preserve features in the low-level layers. The number of decoders is N_c_, where N_c_ is the number of classes to be segmented, and each decoder is designed to segment the domains according to the features of each class. To achieve accurate segmentation of the small-sized ureter, the output resolution was adjusted to match the input resolution, in contrast to the approach utilised by many segmentation architectures such as U-Net, PraNet, and FCB-SwinV2 Transformer, which predict or interpolate a smaller size than the input size. For upsampling in the decoder, the transposed convolution method was utilised to ensure that the output has the same resolution as the input^28^. The final feature map is created by stacking the feature maps created by each decoder.

In the encoder blocks, a residual connection was used to maintain the identity features and resolution of the input and output of each encoder layer. Three, four, six, and three residual blocks were used for each encoder level. Similar to U-Net, a 2 × 2 max pooling layer with stride 2 was used for down sampling. From the first layer to the last layer of the encoder, each layer had 128, 256, 512, and 1024 channels. In the decoder layers, we constructed six, four, and three decoder blocks corresponding to the encoder levels. Like the U-Net, a 2 × 2 up-conv was used to reduce the number of channels. The skip connection was applied from the encoder to the decoder, and each decoder layer had 1024, 512, and 256 channels, respectively.

Deep supervision is used during the training process^29^. The output of each layer of the decoder, reported in binary format, was upscaled to the size of the original image and compared with the ground truth. The deep supervision loss function was calculated by combining the binary cross-entropy and Dice-coefficient loss. A binary cross-entropy loss function was used to distinguish whether each pixel corresponded to the foreground or background, and a Dice coefficient loss function was added to improve the accuracy at the boundaries. The loss function (*Loss*_*DS*_) used for deep supervision in each class *c* and decoder layer *l* can be expressed as:

1 $${Loss}_{DS}\left({x}_{c}, {y}_{c}\right)=BCE\left({x}_{c}, {y}_{c}\right)+1-DICE({x}_{c},{y}_{c})$$

**Figure 5:**
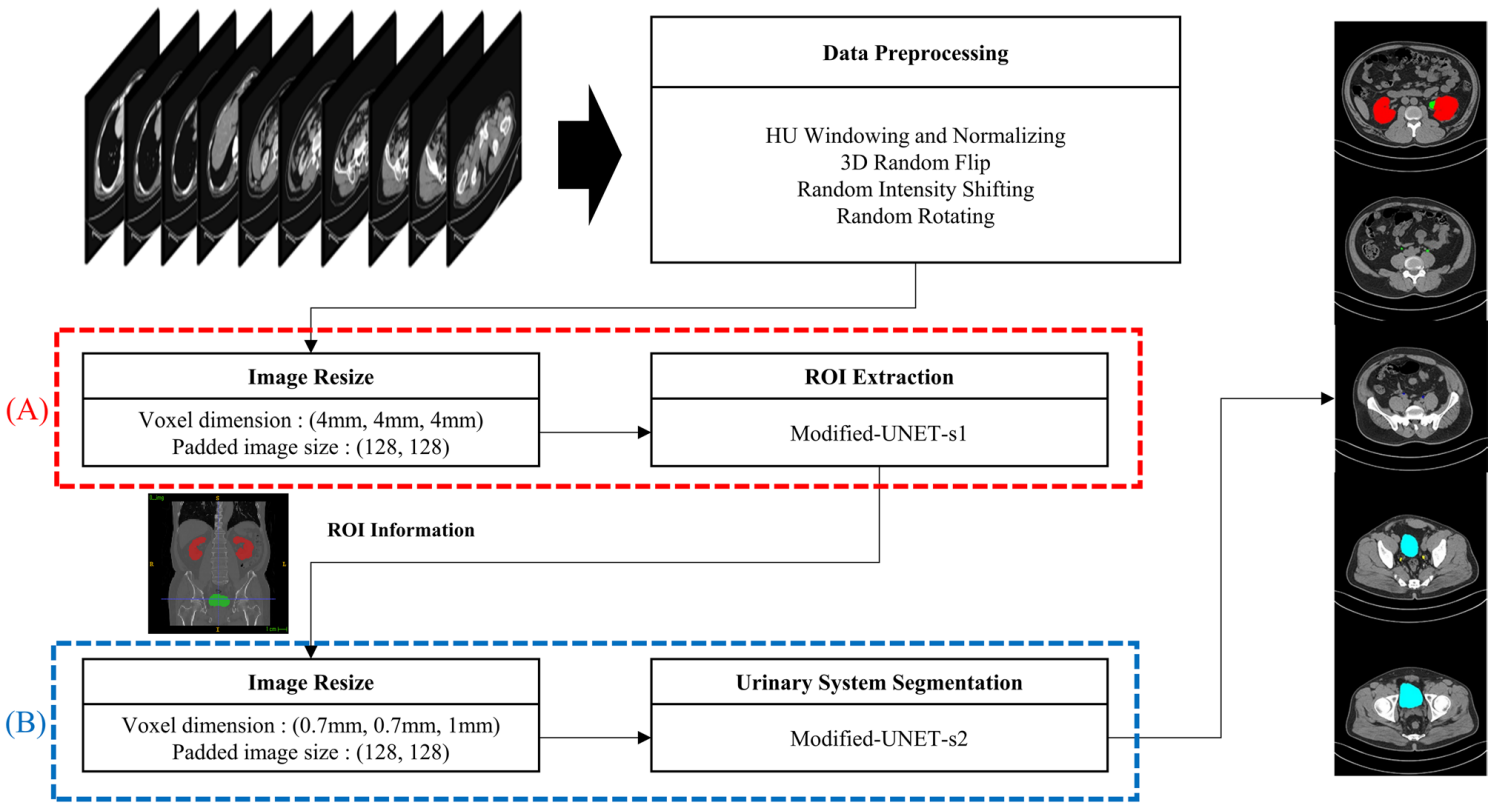
Segmentation process of urinary system on non-contrast abdominopelvic computed tomography images: (**A**) step 1—region of interest extraction, (**B**) step 2—urinary system segmentation. ROI, region of interest.

In this study, urinary system segmentation was performed using a two-step procedure, as shown in Fig. 5. First, the ROI in which the urinary system is estimated to be located is extracted, and then the kidney, ureter and urinary bladder are segmented from the extracted ROI. A modified U-Net was used for steps 1 and 2, and several parameters were modified according to the nature of each step.

### Extraction of ROI

The purpose of ROI extraction is to (1) reduce the possibility of false positives by excluding the part where the urinary system is unlikely to be in the image when considering human anatomy, and (2) reduce the computational cost of the entire process. As the position of the ureter in the 3D space is rarely outside the cuboid, with the two kidneys and urinary bladder as the outer margin, the ROI was estimated only through the position of the two kidneys and urinary bladder without considering the position of the ureter. When extracting the ROI, an extra margin was established, considering the possibility that the urinary system exists in a location partially outside the area suggested by the model prediction. Based on the area with the outer margin determined by the two kidneys and the urinary bladder, areas of 15 mm each in the anterior and posterior directions and 6 mm each in the other directions were additionally extracted.

During the ROI extraction process, the resolution of the image was not important. Therefore, the voxel size was set to 4 mm and the size of the axial input image was set to 128 × 128, which was less than that of the original image, using the bilinear interpolation and padding technique. After the ROI extraction, all CT scans used in the present study were reviewed again to confirm whether the ureter travelled beyond the cuboid determined by the two kidneys and urinary bladder.

### Segmentation of urinary system

During the image segmentation process, the ground truth was composed of the kidney, ureter, and urinary bladder, and the ureter was subdivided into proximal, middle, and distal ureters. For urinary system segmentation, a region corresponding to a determined ROI was extracted from the axial images and the pixel size of the extracted image was set to 0.7 mm, which is the average pixel size of the CT images of the two study institutions. The prediction was made using a sliding window approach with a crop window size of 128 × 128.

Urinary system segmentation was performed in a two-dimensional space, using each axial image as input for training. Random point sampling was used to solve the problem of overfitting and the increase in computational cost that may occur when the entire axial image is sequentially scanned with a sliding window during the learning process. When the ROI extracted from the axial image has a two-dimensional space of H × W, the ‘Cropable Centre Area (CCA)’ in which the centre of a window of size k × k can exist can be defined as follows:

2 $$CCA(I)=\{x, y|\frac{k}{2}\le x<W-\frac{k}{2}, \frac{k}{2}\le y<H-\frac{k}{2}\}$$

During the learning process, a maximum of 10 non-overlapping random points were determined for each epoch within the region where the centre of the window could exist, and the windows with the corresponding centre were used for scanning. Windows using random sampled points from CCA(I) in Eq. (2) are applied differently to each epoch and may cause an augmentation effect in the learning process.

In the validation process of the trained model, the results derived from the windows were combined. The axial image was examined while sliding the window at regular intervals to have an overlap ratio of 0.5, and the result was derived by assembling the probabilities derived for each point from each overlapped window (Supplementary Fig. 1).

As post-processing methods, the argmax operation was utilised for each pixel, identifying the class with the highest probability along the class axis. Additionally, a 50% threshold was applied to these argmaxed pixels, categorising those below 50% probability as background and those above 50% as foreground.

### Statistical analyses

The Dice coefficient was used as a quantitative metric to evaluate the performance of the model created for urinary system segmentation. The Dice coefficient was defined as the ratio of the overlapped area between the segmented image and corresponding ground-truth image to the total dimension of both images. It takes a value between 0 and 1, where 1 means that perfect segmentation has been achieved, and 0 means no overlap between the segmented image and corresponding ground-truth image. When S and G mean the area of the segmented image and corresponding ground-truth image, respectively, the Dice coefficient is defined as follows:

3 $$Dice coefficient=\frac{2(S\cap G)}{\left|S\right|+\left|G\right|}$$

Because of the small size of the datasets, a fivefold cross-validation was performed to evaluate the robustness of the model performance. Finally, the model that showed the highest performance in the fivefold cross validation process was selected as the final model and its performance was measured on a separate test dataset. In addition, the performance of the final model was assessed by comparing it with that of other previously proposed models. Segmentation of the urinary system in each region was performed using several models on the same dataset, and the Dice score was calculated.

The modified U-Net was implemented using PyTorch 1.10.0 for the deep learning framework along with Torchvision 0.11.1 for image and vision tasks, all in Python 3.8.10. The following libraries were utilised for medical image handling: SimpleITK 2.1.0, Pydicom 2.2.0, and Nibabel 3.2.0. Additionally, Pillow 8.0.0 and OpenCV 4.5.4.60 were employed for image processing. All training and validation processes were performed with two NVIDIA RTX 3090 Ti graphics processing units (NVIDIA, Santa Clara, CA, USA).

### Supplementary Information

Supplementary Figure 1.

## Data Availability

The datasets generated and analysed during the current study are not publicly available because permission to share patient data was not granted by the institutional review board, although they are available from the corresponding author upon reasonable request.
