# Supplementary material for: Kidney, ureter, and urinary bladder segmentation based on non-contrast enhanced computed tomography images using modified U-Net
Source: Sci Rep. 2024 Jul 3;14:15325. doi: 10.1038/s41598-024-66045-6 (PMC11222420; doi:10.1038/s41598-024-66045-6)
Supplement: Supplementary file 1 — Supplementary Figure 1. [file 41598_2024_66045_MOESM1_ESM.pdf]

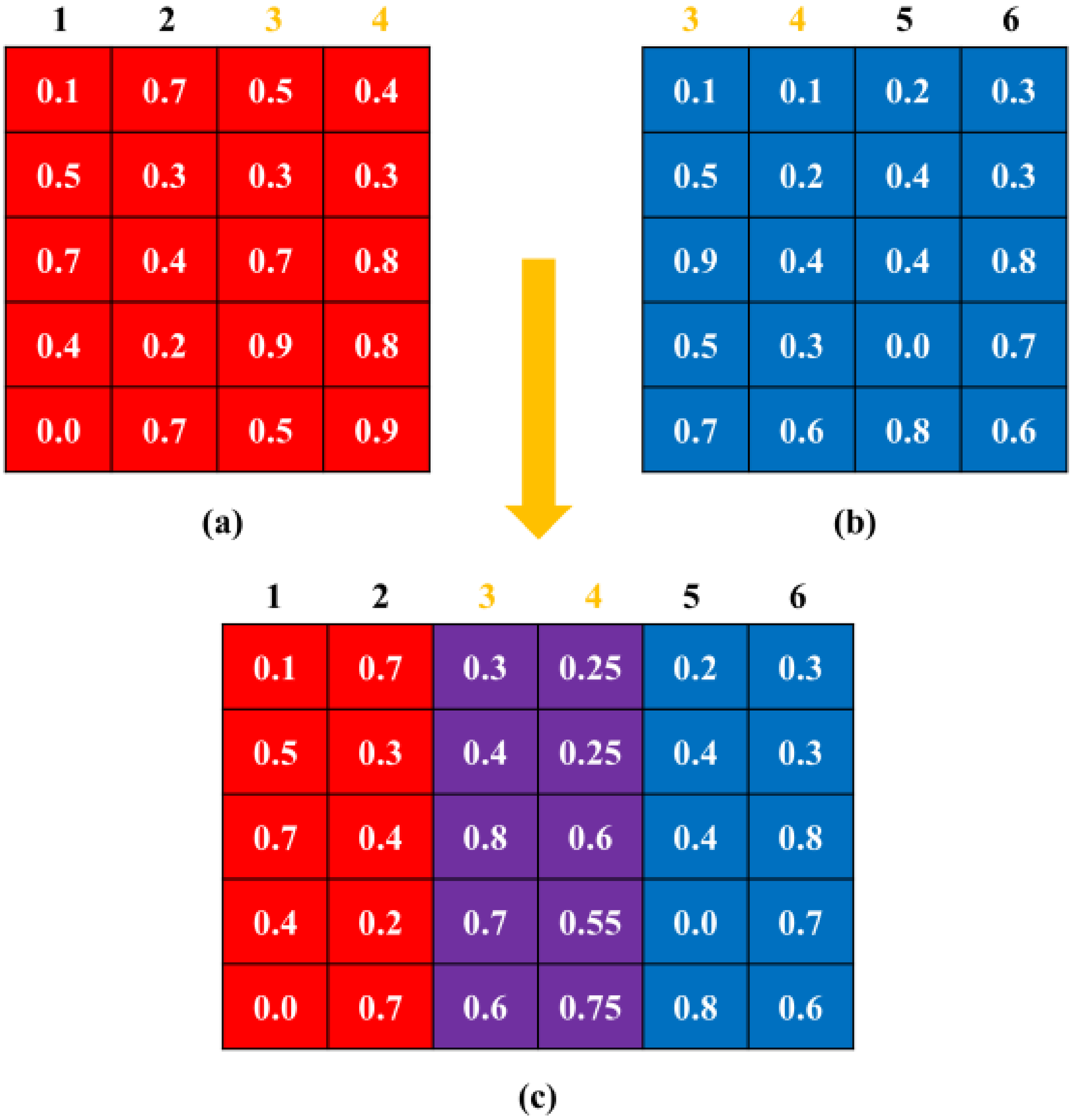

Supplementary Fig. 1. Sliding window ensemble example. Results of the overlapped area were derived by assembling the probabilities defined by each window.
